# Inhibition of Metabolism as a Therapeutic Option for Tamoxifen-Resistant Breast Cancer Cells

**DOI:** 10.3390/cells10092398

**Published:** 2021-09-12

**Authors:** Friederike Steifensand, Julia Gallwas, Gerd Bauerschmitz, Carsten Gründker

**Affiliations:** Department of Gynecology and Obstetrics, University Medicine Göttingen, 37075 Göttingen, Germany; f.steifensand@stud.uni-goettingen.de (F.S.); julia.gallwas@med.uni-goettingen.de (J.G.); gerd.bauerschmitz@med.uni-goettingen.de (G.B.)

**Keywords:** breast cancer, estrogen receptor α, tamoxifen resistance, glutaminolysis, glycolysis

## Abstract

Cancer cells have an increased need for glucose and, despite aerobic conditions, obtain their energy through aerobic oxidation and lactate fermentation, instead of aerobic oxidation alone. Glutamine is an essential amino acid in the human body. Glutaminolysis and glycolysis are crucial for cancer cell survival. In the therapy of estrogen receptor α (ERα)-positive breast cancer (BC), the focus lies on hormone sensitivity targeting therapy with selective estrogen receptor modulators (SERMs) such as 4-hydroxytamoxifen (4-OHT), although this therapy is partially limited by the development of resistance. Therefore, further targets for therapy improvement of ERα-positive BC with secondary 4-OHT resistance are needed. Hence, increased glucose requirement and upregulated glutaminolysis in BC cells could be used. We have established sublines of ERα-positive MCF7 and T47D BC cells, which were developed to be resistant to 4-OHT. Further, glycolysis inhibitor 2-Deoxy-D-Glucose (2-DG) and glutaminase inhibitor CB-839 were analyzed. Co-treatments using 4-OHT and CB-839, 2-DG and CB-839, or 4-OHT, 2-DG and CB-839, respectively, showed significantly stronger inhibitory effects on viability compared to single treatments. It could be shown that tamoxifen-resistant BC cell lines, compared to the non-resistant cell lines, exhibited a stronger reducing effect on cell viability under co-treatments. In addition, the tamoxifen-resistant BC cell lines showed increased expression of proto-oncogene c-Myc compared to the parental cell lines. This could be reduced depending on the treatment. Suppression of c-Myc expression using specific siRNA completely abolished resistance to 4OH-tamoxifen. In summary, our data suggest that combined treatments affecting the metabolism of BC are suitable depending on the cellularity and resistance status. In addition, the anti-metabolic treatments affected the expression of the proto-oncogene c-Myc, a key player in the regulation of cancer cell metabolism.

## 1. Introduction

In human tissue, two estrogen receptors (ERs) can basically be distinguished from one another, ERα and ERβ. Both have the same basic structure but are expressed in different tissues and show different effects. While ERα is mainly found in the breast, ERβ is expressed in bone, endometrium and blood vessels [1]. Tamoxifen acts as a selective estrogen receptor modulator (SERM) on ER and is used in breast cancer cells, in case these express ERα. Tamoxifen is a specific antagonist for ERα and thus shows an antiproliferative effect. Tamoxifen is converted into the active and significantly more effective metabolite 4-OH tamoxifen (4-OHT) via cytochrome P (CYP) in the liver [2]. It binds competitively to ERα, but in such a way that there is a change in conformation resulting in an ER antagonism. A complex is formed with coactivators, leading to an inhibitory effect on EREs and suppresses the ER-dependent transcription. Tamoxifen thus acts as a SERM at the transcriptional level. Of breast cancers, 75% are hormone-sensitive and can therefore be treated with tamoxifen [3]. On ERβ in contrast, tamoxifen has a proliferative effect, which is why side effects arise. In addition to general symptoms (malaise, fatigue, nausea, edema, hot flashes, leukocytopenia and thrombocytopenia), an increased risk of developing endometrial cancer (1%) and development of thromboembolism (1–3%) is known [4,5]. In addition to the occurrence of possible side effects, the development of tamoxifen resistance is a major problem in clinical practice. Primary or secondary tamoxifen resistance can be observed in over 50% of the patients [6]. The exact pathomechanism behind the development of resistance is not yet fully understood. Therefore, it is important to develop new, innovative therapeutic options for this group of patients.

Selectivity is a cornerstone in cancer therapy. In order to achieve effective damage of tumor tissue without side effects, it is important to recognize biological differences between normal cells and tumor cells. Normally, aerobic glycolysis takes place under oxygen conditions and anaerobic glycolysis under oxygen deficiency. In aerobic glycolysis, glucose is metabolized into carbon dioxide, water and ATP via citrate cycle and mitochondrial respiratory chain. Anaerobic glycolysis works through the fermentation of pyruvate into lactate with a significantly lower gain of ATP [7]. Cancer cells compared to healthy cells have an increased need for glucose and, despite aerobic conditions, obtain their energy through aerobic oxidation and lactate fermentation, instead of aerobic oxidation alone [8]. Various explanatory approaches attempt to analyze this atypical behavior of tumor cells [9]. Accordingly, increased glucose uptake should accelerate metabolic pathways and enable faster ATP production, which means that tumors can proliferate more quickly [10,11]. In addition, the resulting acidotic milieu should enable tumors to grow invasively [12]. With glycolysis inhibition by 2-Deoxy-D-Glucose (2-DG), it is possible to intervene in this Warburg effect. A second selective property of cancers is the importance of glutaminolysis. Glutamine is an essential amino acid. During glutaminolysis, it is metabolized to glutamate, aspartate, CO_2_, pyruvate, lactate, alanine and citrate [13]. After being absorbed into the cell, the enzyme glutaminase converts it into glutamate and ammonium (NH^4+^). In the next step, glutamate dehydrogenase turns glutamate into α-ketoglutarate, which then can feed the citric acid cycle. Alternatively, glutamate can be converted by alanine aminotransferase or by aspartate aminotransferase into alanine and aspartate, which are essential for nucleic acid synthesis. Malate produced in the intermediate step of the citric acid cycle can be converted into pyruvate or lactate. As part of glutaminolysis, this opens up another process of energy generation in addition to glycolysis [14]. It has already been observed that tumor cells have an increased glutamine metabolism in addition to increased glycolysis [15,16]. The benefit for tumor cells lies in generation of additional energy and in an increased nucleic acid and serine synthesis through glutamate and aspartate, based on glutamine. This enables proliferative growth of tumor cells [15].

This knowledge represents a potential therapeutic target in the way that glutaminolysis is inhibited. The glutaminase inhibitor CB-839 interferes with the first step of glutaminolysis by selectively and irreversibly binding the enzyme glutaminase [17]. Increased glutaminase has already been demonstrated in glioma [18], non-small cell lung cancer [19], pancreatic cancer [20] and triple-negative breast cancer cells, among others [21]. Recently, it was shown that glutaminase levels in breast cancer patients correlated with increased tumor grading and staging [22]. In addition, an increase in glutaminase expression was demonstrated in tumors without expression of ER and progesterone receptor (PR) [22]. The effect of glutaminase inhibitor CB-839 could be demonstrated in triple-negative breast cancer cells [17]. Clinical phase 1 and 2 studies are currently ongoing with regard to monotherapy and combination therapy with CB-839 in solid tumors, including triple-negative breast cancer (NCT02071862, NCT03057600, NCT03875313) but not ERα-positive breast cancer [23]. In various studies, 4-OHT, 2-DG and CB-839 were able to individually show an antiproliferative effect on ERα-positive (4-OHT) and ERα-negative (2-DG, CB-839) breast cancer cells [5,17,24]. However, it is not known to what extent a combination of these drugs has an antiproliferative effect on ERα-positive breast cancer cells and how this behaves in comparison to corresponding tamoxifen-resistant cells. This work examined whether therapy with low-dose 4-OHT can be improved by anti-metabolism therapy using 2-DG and/or CB-839 and whether differences in tamoxifen-resistant cells can be observed. Since the oncogenic protein c-Myc was shown to play a main role in the regulation of cancer cell metabolism, including glycolysis and glutaminolysis [25], we further investigated whether the various anti-metabolic treatments influence the expression of c-Myc.

## 2. Materials and Methods

### 2.1. Cell Lines and Culture Conditions

The human breast cancer cell lines MCF7 and T47D were obtained from the American Type Culture Collection (Manassas, VA, USA). To guarantee the identity of the cell lines over the years, the cells were expanded after purchase and aliquots were stored in liquid nitrogen. Every half-year, new frozen stock was opened and expanded to carry out the experiments. Tamoxifen-resistant sublines MCF7-TR and T47D-TR were developed as described in detail [26], and the medium concentration of 4-OH-tamoxifen (4-OHT; Sigma, Deisenhofen, Germany) was 125 nM. The cells were cultured at 37 °C in a humidified atmosphere of 5% CO_2_ in air as previously described [26].

### 2.2. Drugs

4-hydroxytamoxifen (4-OHT) and 2-Deoxy-D-glucose (2-DG) were purchased from Sigma. Glutaminase inhibitor CB-839 was purchased from Selleckchem (München, Germany).

### 2.3. Viability Assay

A total of 12,500 cells per well were plated into 96-well plates (Falcon, Corning, NY, USA) in 100 μL Dulbecco’s Modified Eagle’s Medium/5% fetal calf serum (FCS, Biochrom, Berlin, Germany) without phenol red, 2 mM glutamine, 50 U/mL penicillin/streptomycin, 2.5 μg/mL amphotericin B and 1% non-essential amino acids. After cell attachment, 100 μL medium, 100 μL 4-OHT/medium solution, 100 μL 2-DG/medium solution, 100 μL CB-839/medium solution or 100 μL solution with combination treatments were added to the wells and incubated for 96 h at 37 °C, 5% CO_2_. Final concentrations of 4-OHT, 2-DG, CB-839 and the combinations of the agents are given in the results section. Cell number was determined by a colorimetric assay using Alamar Blue (BioRad, Puchheim, Germany). The optical density (OD) of the reduced dye was assessed at 570 versus 630 nm after 4 h at 37 °C.

### 2.4. Mitochondrial Membrane Potential

Cells were treated for 48 h with or without 4-OHT, 2-DG, CB-839 or the combinations of the agents. Then they were washed once with PBS and mitochondrial membrane potential was measured using the JC-1 mitochondrial membrane potential detection kit according to the instructions of the manufacturer (Biotium, Köln, Germany).

### 2.5. c-Myc Expression

In Western Blot analysis, cells were lysed in cell lytic M buffer (Sigma) supplemented with 0.1% phosphatase-inhibitor (Sigma) and 0.1% protease-inhibitor (Sigma). Isolated proteins (40 µg) were fractioned using 12% SDS gel and electro-transferred to a polyvinylidene difluoride membrane (Merck Millipore, Cork, Ireland). Primary antibodies against c-Myc 1:10,000 (Abcam, Cambridge, UK) and GAPDH 1:2000 (Cell Signaling, Danvers, MA, USA) were used. The membrane was washed and incubated in horseradish peroxidase-conjugated secondary antibody (GE Healthcare, Buckinghamshire, UK). Antibody-bond protein bands were assayed using a chemiluminescent luminol enhancer solution (Cyanagen, Bologna, Italy). For visualization and quantification, the bands were scanned using a C-DiGit Blot Scanner (LI-COR Biosciences, Lincoln, NE, USA).

### 2.6. Small Interfering RNA Transfection

Breast cancer cell lines MCF7-EMT (5 × 10^5^ cells/mL) and T47D-EMT (2.5 × 10^5^ cells/mL) were seeded in 2 mL of MEM with 10% FBS (-P/S) in 25 cm^2^ cell culture flask. The cells were transiently transfected with siRNA specific to c-Myc (sc-29226 c-Myc-specific siRNA; Santa Cruz Biotechnology, Dallas, TX, USA) in OPTI-MEM I medium (Gibco, Carlsbad, CA, USA) with a siRNA transfection reagent (sc-29528; Santa Cruz Biotechnology, Dallas, TX, USA). A non-targeting siRNA was used as control (sc-37007 control-A; Santa Cruz Biotechnology, Dallas, TX, USA). After an incubation period of 6 h, MEM supplemented with 20% FBS and 20% penicillin/streptomycin was added.

### 2.7. Statistical Analysis

All experiments were repeated at least three times with different passages of the respective cell lines. The data were tested for significant differences by one-way analysis of variance followed by Tukey’s multiple comparisons test for comparison of individual groups after a Bartlett test had shown that variances were homogenous using GraphPad Prism 7 software (GraphPad Software, San Diego, CA, USA).

## 3. Results

We analyzed, whether the therapy of ERα-positive human breast cancer cell lines MCF7 and T47D with 4-OHT can be optimized by an anti-metabolism therapy using 2-DG and/or CB-839 and whether differences to the corresponding tamoxifen-resistant sublines MCF7-TR and T47D-TR could be observed.

### 3.1. Effects on Cell Viability

#### 3.1.1. Single Treatments

First, the effects of 4-OHT, 2-DG and CB-839 on cell viability were determined separately on the cell lines MCF7, MCF7-TR, T47D and T47D-TR using three different doses of each drug.

**Figure 1:**
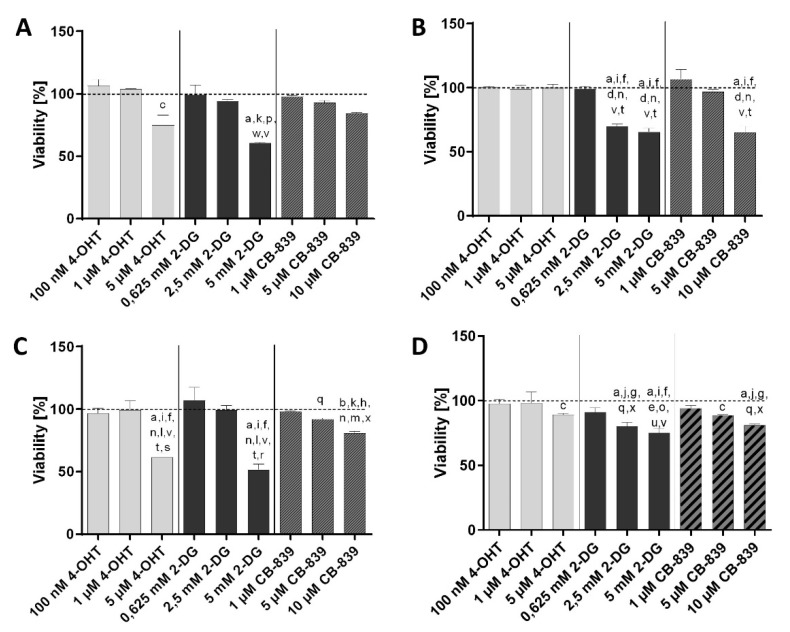
Viability of human breast cancer cell lines MCF7 (**A**), T47D (**C**) and their tamoxifen-resistant sublines MCF7-TR (**B**) and T47D-TR (**D**) after treatment with or without 4-hydroxytamoxifen (4-OHT), inhibitor 2-Deoxy-D-Glucose (2-DG) or glutaminase inhibitor CB-839. Columns represent means ± SEM of data obtained from three independent experiments in three different passages of the cell lines. a, *p* < 0.0001 vs. control; b, *p* < 0.001 vs. control; c, *p* < 0.05 vs. control; d, *p* < 0.0001 vs. 5 µM 4-OHT; e, *p* < 0.01 vs. 5 µM 4-OHT; f, *p* < 0.0001 vs. 1 µM 4-OHT; g, *p* < 0.001 vs. 1 µM 4-OHT; h, *p* < 0.01 vs. 1 µM 4-OHT; i, *p* < 0.0001 vs. 100 nM 4-OHT; j, *p* < 0.001 vs. 100 nM 4-OHT; k, *p* < 0.05 vs. 100 nM 4-OHT; l, *p* < 0.0001 vs. 2.5 mM 2-DG; m, *p* < 0.01 vs. 2.5 mM 2-DG; n, *p* < 0.0001 vs. 0.625 mM 2-DG; o, *p* < 0.001 vs. 0.625 mM 2-DG; p, *p* < 0.01 vs. 0.625 mM 2-DG; q, *p* < 0.05 vs. 0.625 mM 2-DG; r, *p* < 0.0001 vs. 10 µM CB-839; s, *p* < 0.01 vs. 10 µM CB-839; t, *p* < 0.0001 vs. 5 µM CB-839; u, *p* < 0.01 vs. 5 µM CB-839; v, *p* < 0.0001 vs. 1 µM CB-839; w, *p* < 0.001 vs. 1 µM CB-839; x, *p* < 0.01 vs. 1 µM CB-839 (ANOVA followed by Tukey’s multiple comparisons test).

The MCF7 cell line (Figure 1A, Supplementary Table S1) showed a significant reduction in cell viability after treatment with the highest tamoxifen concentration (5 µM) compared to the untreated control, while no effect was observed with the tamoxifen-resistant cell line. After treatment with 2.5 mM 2-DG, the tamoxifen-resistant MCF7-TR subline (Figure 1B, Supplementary Table S1) showed a significant reduction of viability in comparison to the control, the 4-OHT treatments, the lowest 2-DG concentration (0.625 mM) and 1 µM as well as 5 µM concentrations of CB-839. The viability of MCF7 cells (Figure 1A, Supplementary Table S1) was significantly reduced after treatment with the highest concentration of 2-DG (5 mM) compared to the untreated control, the lowest 4-OHT concentration (100 nM), the lowest 2-DG concentration (0.625 mM) and both lower concentrations of CB-839 (1 µM, 5 µM). The tamoxifen-resistant MCF7-TR subline (Figure 1B, Supplementary Table S1) also showed a significant reduction in viability after treatment with 5 mM 2-DG in comparison to the control, all 4-OHT treatments, the lowest 2-DG treatment (0.625 mM) and both lower CB-839 treatments (1 µM, 5 µM). After treatment of the MCF7-TR cells (Figure 1B, Supplementary Table S1) with the highest concentration of glutaminase inhibitor CB-839 (10 µM), the viability was significantly reduced compared to the control, all 4-OHT treatments, the lowest 2-DG treatment and both lower CB-839 concentrations. The parental MCF7 cell line showed no significant inhibition of viability under any of the treatments with CB-839.

Under therapy with 4-OHT, the T47D cell line (Figure 1C, Supplementary Table S1) showed a significant reduction of viability at the highest concentration (5 µM), compared to the control and the treatments with both lower 4-OHT concentrations, both lower 2-DG doses and the CB-839 treatments.

There was also a significant inhibition of viability with the T47D-TR subline detectable compared to the untreated control (Figure 1D, Supplementary Table S1). After treatment with 2-DG, the T47D-TR cell line (Figure 1D, Supplementary Table S1) showed a significant reduction in viability at 2.5 mM concentration compared to the untreated control, both lower concentrations of 4-OHT, the lowest concentration of 2-DG and the lowest concentration of CB-839. At 5 mM concentration of 2-DG, viability was significantly reduced compared to the untreated control, all 4-OHT treatments, the lowest 2-DG treatment and both lower CB-839 treatments. In the parental T47D cell line (Figure 1C, Supplementary Table S1), the highest concentration of 2-DG (5 mM) had a significant inhibitory effect on viability compared to the control, both lower 4-OHT treatments, both lower 2-DG treatments and the CB-839 treatments.

The treatment of the T47D cell line (Figure 1C, Supplementary Table S1) with 5 μM and 10 µM concentrations of CB-839 showed a significant reduction in viability compared to the lowest 2-DG concentration. With the T47D-TR cell line (Figure 1D, Supplementary Table S1), significant inhibition of viability was observed under treatment with 5 μM CB-839 in comparison to the untreated control. Treatment with 10 µM CB-839 also resulted in a significant reduction in viability in comparison to the control, both lower 4-OHT treatments and the lowest treatment with 2-DG or CB-839.

#### 3.1.2. Combination Treatments

**Figure 2:**
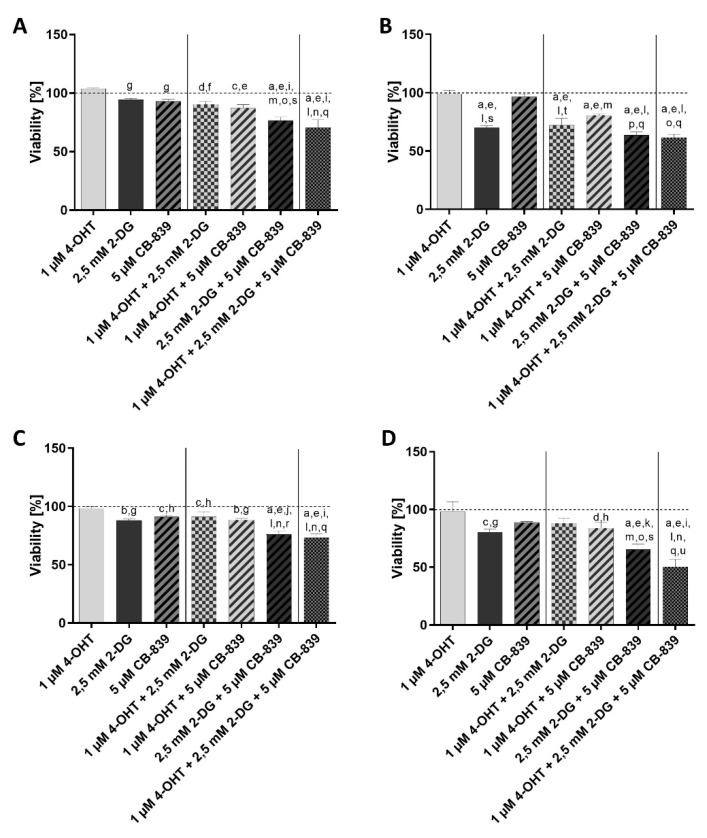
Viability of human breast cancer cell lines MCF7 (**A**), T47D (**C**) and their tamoxifen-resistant sublines MCF7-TR (**B**) and T47D-TR (**D**) after treatment with or without 4-OHT, 2-DG or CB-839 or different combinations. Columns represent means ± SEM of data obtained from three independent experiments in three different passages of the cell lines. a, *p* < 0.0001 vs. control; b, *p* < 0.001 vs. control; c, *p* < 0.01 vs. control; d, *p* < 0.05 vs. control; e, *p* < 0.0001 vs. 1 µM 4-OHT; f, *p* < 0.001 vs. 1 µM 4-OHT; g, *p* < 0.01 vs. 1 µM 4-OHT; h, *p* < 0.05 vs. 1 µM 4-OHT; i, *p* < 0.0001 vs. 2.5 mM 2-DG; j, *p* < 0.001 vs. 2.5 mM 2-DG; k, *p* < 0.05 vs. 2.5 mM 2-DG; l, *p* < 0.0001 vs. 5 µM CB-839; m, *p* < 0.001 vs. 5 µM CB-839; n, *p* < 0.0001 vs. 1 µM 4-OHT + 2.5 mM 2-DG; o, *p* < 0.001 vs. 1 µM 4-OHT + 2.5 mM 2-DG; p, *p* < 0.05 vs. 1 µM 4-OHT + 2.5 mM 2-DG; q, *p* < 0.0001 vs. 1 µM 4-OHT + 5 µM CB-839; r, *p* < 0.001 vs. 1 µM 4-OHT + 5 µM CB-839; s, *p* < 0.01 vs. 1 µM 4-OHT + 5 µM CB-839; t, *p* < 0.05 vs. 1 µM 4-OHT + 5 µM CB-839; u, *p* < 0.05 vs. 2.5 mM 2-DG + 5 µM CB-839 (ANOVA followed by Tukey’s multiple comparisons test).

Treatment with the combination of 4-OHT and 2-DG resulted in a significant reduction in the viability of MCF7 cells (Figure 2A, Supplementary Table S2) compared to the control and the 4-OHT therapy, just as with the combination of 4-OHT and CB-839. The combination treatment with 2-DG and CB-839 and the combination of the three compounds 4-OHT, 2-DG and CB-839 showed a significant reduction in viability compared to the control, the single treatment with 4-OHT, 2-DG or CB-839 alone and the combination therapies with 4-OHT/2-DG and 4-OHT/CB-839.

The MCF7-TR cell line (Figure 2B, Supplementary Table S2) showed a significant reduction in viability under the treatment with 2-DG alone as well as under the treatment with the combination of 4-OHT and 2-DG, in comparison to the untreated control, treatment with 4-OHT or CB-839 alone, as well as the combination treatment of 4-OHT/CB-839. The combination treatment of 4-OHT and CB-839 showed a significantly stronger inhibition of viability compared to the control and the 4-OHT and CB-839 treatments alone.

During the treatment with the combination of 2-DG and CB-839, a significantly stronger viability-inhibiting effect was observed compared to the treatments with 4-OHT, CB-839, 4-OHT/2-DG and 4-OHT/CB-839. Similarly, the combination of all three drugs showed a significantly stronger inhibition of viability compared to the treatments with 4-OHT, CB-839, 4-OHT/2-DG and 4-OHT/CB-839.

The single treatments of T47D cells (Figure 2C, Supplementary Table S2) with 2-DG and CB-839 as well as the combination treatments with 4-OHT/2-DG and 4-OHT/CB-839 showed a significant reduction in viability compared to the untreated control and the 4-OHT therapy alone.

Treatment of T47D cells (Figure 2C, Supplementary Table S2) with the combination of the two metabolism inhibitors 2-DG and CB-839 and the triple combination of all three drugs resulted in a significantly stronger viability-inhibiting effect compared to all single treatments and the combinations 4-OHT/2-DG and 4-OHT/CB-839.

Treatment of T47D-TR cells (Figure 2D, Supplementary Table S2) with 2-DG alone and the combination of 4-OHT and CB-839 resulted in a significant viability-inhibiting effect compared to the untreated control and the treatment with 4-OHT alone. Treatment of T47D-TR cells with the combination of the two metabolism inhibitors resulted in significantly stronger inhibition of viability compared to all three single treatments and the two other double combinations 4-OHT/2-DG and 4-OHT/CB-839. The triple treatment of T47D-TR cells was, compared to all single and double treatments, significantly more effective.

### 3.2. Induction of Apoptosis

**Figure 3:**
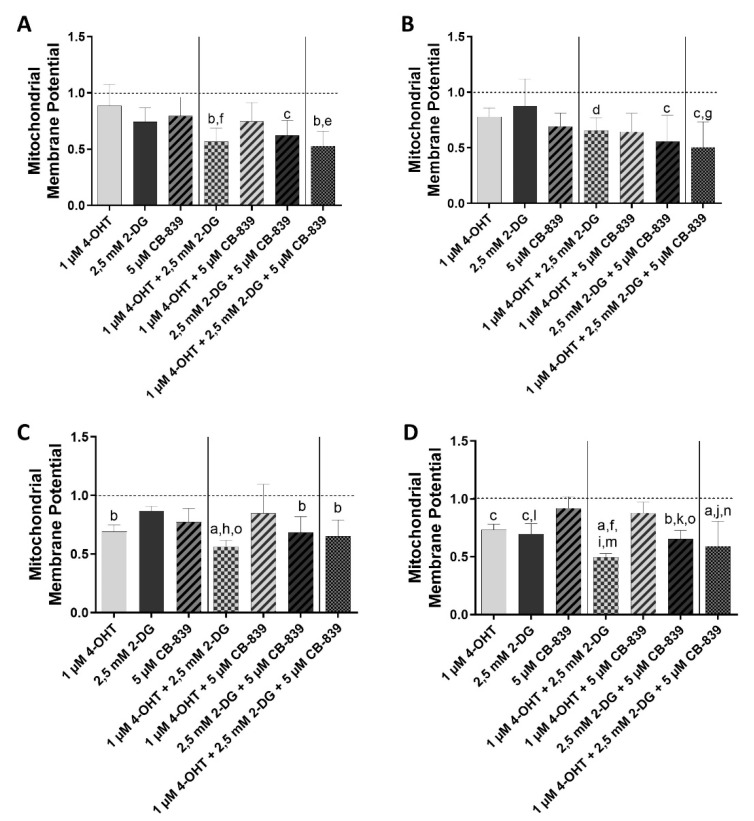
Mitochondrial membrane potential of human breast cancer cell lines MCF7 (**A**), T47D (**C**) and their tamoxifen-resistant sublines MCF7-TR (**B**) and T47D-TR (**D**) after treatment with or without 4-OHT, 2-DG or CB-839 or different combinations. Columns represent means ± SEM of data obtained from three independent experiments in three different passages of the cell lines. a, *p* < 0.0001 vs. control; b, *p* < 0.001 vs. control; c, *p* < 0.01 vs. control; d, *p* < 0.05 vs. control; e, *p* < 0.01 vs. 1 µM 4-OHT; f, *p* < 0.05 vs. 1 µM 4-OHT; g, *p* < 0.05 vs. 5 mM 2-DG; h, *p* < 0.001 vs. 2.5 mM 2-DG; i, *p* < 0.0001 vs. 5 µM CB-839; j, *p* < 0.001 vs. 5 µM CB-839; k, *p* < 0.01 vs. 5 µM CB-839; l, *p* < 0.05 vs. 5 µM CB-839; m, *p* < 0.0001 vs. 1 µM 4-OHT + 5 µM CB-839; n, *p* < 0.01 vs. 1 µM 4-OHT + 5 µM CB-839; o, *p* < 0.05 vs. 1 µM 4-OHT + 5 µM CB-839 (ANOVA followed by Tukey’s multiple comparisons test).

The combination therapy of MCF7 cells (Figure 3A, Supplementary Table S3) with 4-OHT and 2-DG showed significant downregulation of the mitochondrial membrane potential compared to the control and the single treatment with 4-OHT. The combination treatment consisting of the two metabolism inhibitors 2-DG and CB-839 showed a significantly stronger reduction in the mitochondrial membrane potential compared to the untreated control. The combination treatment of all three drugs resulted in a significantly stronger inhibition of the mitochondrial membrane potential in comparison to the untreated control and the 4-OHT therapy alone.

Treatment of the MCF7-TR cell line (Figure 3B, Supplementary Table S3) with the combinations of 4-OHT and 2-DG or the metabolism inhibitors 2-DG and CB-839 resulted in a significant reduction of mitochondrial membrane potential in comparison to the untreated control. The triple combination, consisting of 4-OHT, 2-DG and CB839, resulted in a significant reduction in the mitochondrial membrane potential compared to the untreated control and the 2-DG therapy alone.

After the treatment of T47D cells (Figure 3C, Supplementary Table S3) with 4-OHT alone or the combination with 2-DG/CB-839 or with 4-OHT/2-DG/CB-839, the mitochondrial membrane potential was significantly lower compared to the untreated control. Treatment of T47D cells with the combination of 4-OHT and 2-DG resulted in a significant reduction in mitochondrial membrane potential as compared to the control, the single 2-DG treatment and the combination treatment 4-OHT/CB-839.

The T47D-TR cell line (Figure 3D, Supplementary Table S3) showed a significant reduction in mitochondrial membrane potential after treatment with 4-OHT alone in comparison to the untreated control. There was also a significant reduction under therapy with 2-DG alone as compared to the untreated control and CB-839 alone.

The combination treatment 4-OHT/2-DG of T47D-TR cells (Figure 3D, Supplementary Table S3) led to a significant reduction of the mitochondrial membrane potential as compared to the control, the single treatments with 4-OHT or with CB-839 and to the combination treatment with 4-OHT/CB-839. The combination treatment consisting of the two metabolism inhibitors 2-DG and CB-839 resulted in a significant reduction of the mitochondrial membrane potential in comparison to the control, the CB-839 treatment and the 4-OHT/CB-839 combination treatment. Treatment of T47D-TR cells with the combination of 4-OHT, 2-DG and CB-839 led also to a significant reduction of the mitochondrial membrane potential compared to the control, the CB-839 treatment and the 4-OHT/CB-839 combination treatment.

### 3.3. c-Myc

**Figure 4:**
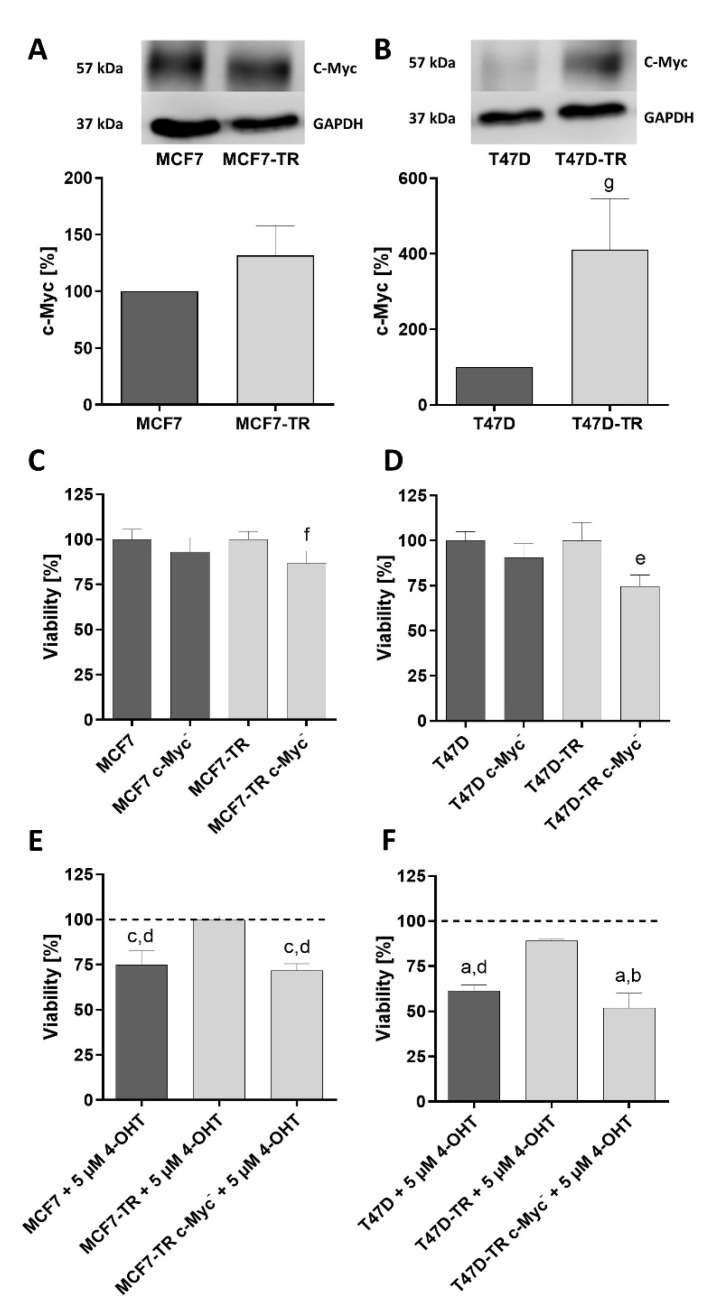
Comparison of c-Myc protein expression in human breast cancer cell lines MCF7 (**A**) and T47D (**B**) and their respective tamoxifen-resistant sublines. Viability of human breast cancer cell lines MCF7 (**C**), T47D (**D**) and their tamoxifen-resistant sublines MCF7-TR (**C**) and T47D-TR (**D**) after c-Myc suppression using specific siRNA. Effect of c-Myc knock down on tamoxifen efficacy on the viability of human breast cancer cell lines MCF7 (**E**), T47D (**F**) and their tamoxifen-resistant sublines MCF7-TR (**E**) and T47D-TR (**F**). Columns represent means ± SEM of data obtained from three independent experiments in three different passages of the cell lines. a, *p* < 0.0001 vs. control; b, *p* < 0.0001 vs. TR cells treated with tamoxifen; c, *p* < 0.001 vs. control; d, *p* < 0.001 vs. TR cells treated with tamoxifen; e, *p* < 0.0001 vs. T47D-TR control; f, *p* < 0.01 vs. MCF7-TR control (ANOVA followed by Tukey’s multiple comparisons test); g, *p*< 0.05 vs. T47D (unpaired *t*-test).

In the next step, we analyzed the role of the proto-oncogene c-Myc during the treatments with 4-OHT, 2-DG, CB-839 and their combinations. First, it was checked to what extent c-Myc was expressed by the four untreated cell lines MCF7, MCF7-TR, T47D and T47D-TR in comparison to one another (Figure 4A,B). The tamoxifen resistant sublines showed a stronger c-Myc expression compared to their parental cell lines, although this was not significant in the MCF7-TR cell line compared to the parental MCF7 cell line. However, there was a significantly stronger c-Myc expression in the T47D-TR cell line compared to the parental T47D cell line (T47D-TR, 411.60 ± 77.82%, *p* < 0.05 vs. T47D).

To confirm the impact of c-Myc suppression on MCF7, MCF7-TR, T47D and T47D-TR breast cancer cells, we assessed whether knock down of c-Myc expression using specific siRNA leads to reduced viability (Figure 4C,D, Supplementary Table S4). Suppression of c-Myc expression in MCF7 (Figure 4C, Supplementary Table S4) and T47D (Figure 4D, Supplementary Table S4) cells resulted in a slightly reduced viability, while decreased expression of c-Myc in the tamoxifen-resistant sublines MCF7-TR (Figure 4C, Supplementary Table S4) and T47D-TR (Figure 4D, Supplementary Table S4) resulted in a significantly reduced viability. Next, we investigated the impact of c-Myc suppression using specific siRNA on tamoxifen resistance (Figure 4E,F, Supplementary Table S4). After knock down of c-Myc expression, treatment of the tamoxifen-resistant cell lines MCF7-TR and T47D-TR with 4-OHT resulted in a comparable reduction of viability as compared with the non-resistant parent lines MCF7 and T47D.

**Figure 5:**
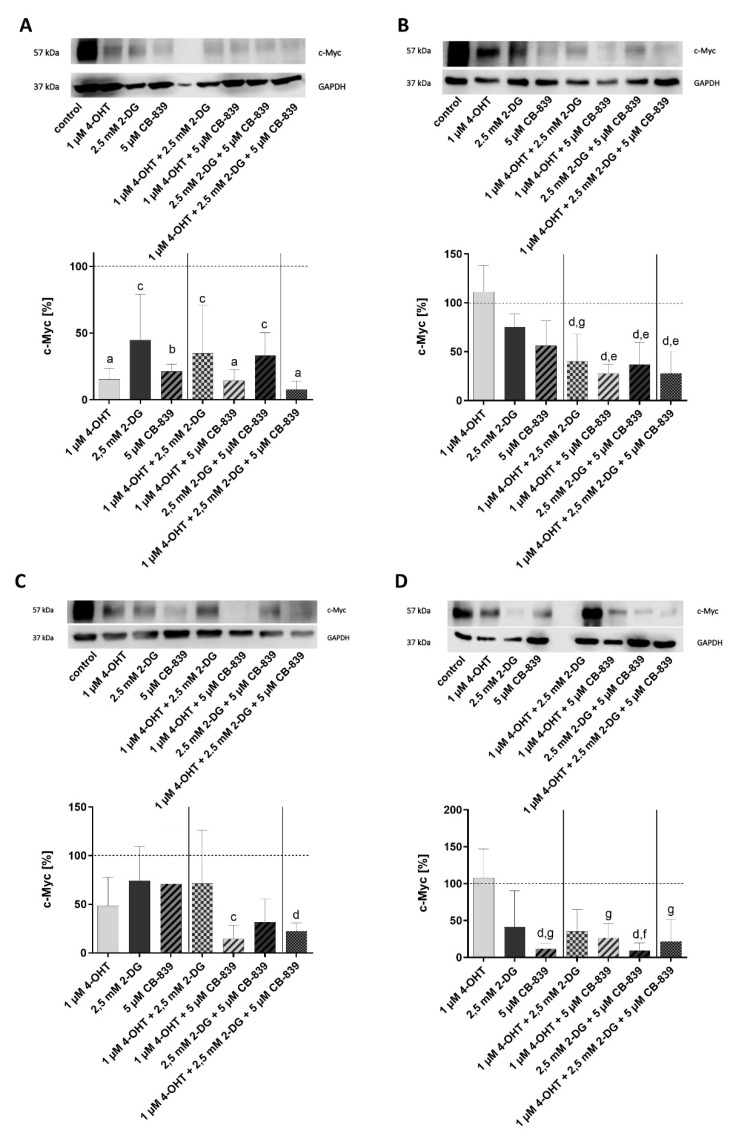
Protein expression of c-Myc in human breast cancer cell lines MCF7 (**A**), T47D (**C**) and their tamoxifen-resistant sublines MCF7-TR (**B**) and T47D-TR (**D**) after treatment without or with 4-OHT, 2-DG or CB-839 or different combinations. Columns represent means ± SEM of data obtained from three independent experiments in three different passages of the cell lines. a, *p* < 0.0001 vs. control; b, *p* < 0.001 vs. control; c, *p* < 0.01 vs. control; d, *p* < 0.05 vs. control; e, *p* < 0.001 vs. 1 µM 4-OHT; f, *p* < 0.01 vs. 1 µM 4-OHT; g, *p* < 0.05 vs. 1 µM 4-OHT (ANOVA followed by Tukey’s multiple comparisons test).

In the next step, we examined the effects of the treatments on the expression of c-Myc. Significant downregulation of c-Myc in the MCF7 cell line (Figure 5A, Supplementary Table S5) was demonstrated under the different treatments compared to the control. Significant downregulation of c-Myc in the MCF7-TR cell line (Figure 5B, Supplementary Table S5) could be shown under the combination treatments in comparison to the control and the 4-OHT therapy alone. In the T47D cell line (Figure 5C, Supplementary Table S5), a significant downregulation of c-Myc has also been observed compared to the untreated control. In the case of the T47D-TR cell line (Figure 5D, Supplementary Table S5), it was found that treatment with CB-839 alone and the combination treatment 2-DG/CB-839 resulted in a downregulation of the c-Myc compared to the control and the 4-OHT treatment alone. The combination treatment, consisting of 4-OHT and CB-839, as well as the combination treatment, consisting of all three active drugs, resulted in significant down-regulation of c-Myc compared to the 4-OHT therapy alone.

## 4. Discussion

The main question of this work was whether the therapy with low-dose 4-OHT can be optimized by an anti-metabolistic therapy using 2-DG and/or CB-839 and whether differences compared to the tamoxifen-resistant sublines could be observed. In addition, the effects of these treatments on the expression of c-Myc should be investigated.

It could be shown, that therapy with 4-OHT resulted in a dose-dependent reduction of viability of both parental, ERα-positive breast cancer cell lines. Treatment of the tamoxifen-resistant sublines showed no or a less pronounced effect on viability. The fact, that treatment of the two sublines with the highest dose of 4-OHT resulted in a significant inhibitory effect on viability suggests that secondary anti-estrogen receptor resistance was not fully developed at this point. 2-DG also led to a dose-dependent reduction in viability, both in the tamoxifen-sensitive and in the tamoxifen-resistant cell lines. In comparison, it could be observed that the tamoxifen-resistant cell lines in both cases showed greater inhibition of viability compared to their parental cell lines at a lower concentration (2.5 mM 2-DG). At a higher concentration (5 mM 2-DG), interestingly, the effect on the parental cell lines was stronger compared to their tamoxifen-resistant cell lines.

A dose-dependent profile of action could be shown under therapy with CB-839 in all four cell lines. However, compared to its parental cell line MCF7, the tamoxifen-resistant cell line MCF7-TR showed a stronger inhibition of viability under the highest CB-839 concentration (10 µM CB-839). The two cell lines T47D and T47D-TR behaved similarly with regard to the action profile. The strongest effects could be achieved with the MCF7 cell line treated with the highest concentrations of 4-OHT or 2-DG. In comparison, the associated tamoxifen-resistant cell line showed the strongest effect under the highest concentrations of the metabolism inhibitors 2-DG and CB-839. The T47D cell line presented the strongest inhibitory effects on cell viability under treatment with the highest concentrations of 4-OHT and 2-DG, whereas the associated tamoxifen-resistant cell line showed the strongest inhibitory effect among the highest concentrations of the metabolism inhibitors 2-DG and CB-839. This initially resulted in the observation that the parental cell lines MCF7 and T47D had the greatest effects under the 4-OHT and 2-DG therapy compared to the other treatments. In contrast, the two tamoxifen-resistant cell lines MCF7-TR and T47D-TR presented strong inhibitions of viability under treatment with the metabolism inhibitors 2-DG and CB-839. In summary, 2-DG could be evaluated as an effective treatment for all four cell lines. 4-OHT showed little or no efficacy in the tamoxifen-resistant cell lines. Here, CB-839 proved to be effective.

Gross et al. showed that CB-839 has a proliferation-inhibitory effect on breast cancer cells from different subtypes, but not on the T47D cell line at low concentrations of 1 µM [17]. This could be shown consistently in this work also in lower concentrations of CB-839 (1 µM and 5 µM), but an active effect could be demonstrated with higher CB-839 concentrations (10 µM). Aft et al. showed an inhibition of cell growth in breast cancer cells under therapy with 2-DG at a concentration of 4 mM [27]. These results confirm the corresponding data of our work. Leung et al. found that tamoxifen-resistant cell lines lead to increased glycolysis and decreased sensitivity of cytotoxic drugs [28]. Based on this presumption, it can be assumed that the tamoxifen-resistant cell lines should show an improved response to an anti-glycolysis therapy.

Our data confirm this presumption to the extent that the parental, non-resistant cell lines MCF7 and T47D only showed a significant reduction in viability above 5 mM 2-DG, the tamoxifen-resistant cell lines MCF7-TR and T47D-TR already below concentrations of 2.5 mM 2-DG. However, in both cell lines, the tamoxifen-resistant cell lines showed a weaker inhibition at the highest concentration (5 mM 2-DG) compared to their parental lines. Subsequently, the effects of the various treatments in combination were examined in more detail and compared in a viability assay. In the case of the MCF7 cell line, the combination treatment of the two metabolism inhibitors, as well as the triple combination, showed the strongest viability inhibition compared to the other treatment alternatives. The associated tamoxifen-resistant cell line showed, in addition to an already strong inhibition of viability under the 2-DG therapy alone, a comparatively strong inhibition by the combined metabolism inhibitors and by the triple combination. In principle, the viability inhibition of the tamoxifen-resistant cell line MCF7-TR was more pronounced than of the parental cell line MCF7. The T47D cell line reacted under the combined metabolism inhibition as well as the triple combination most strongly in a viability-inhibiting manner in contrast to the other treatments. In comparison, the T47D-TR cell line showed the strongest viability-inhibiting effects among the triple combination. Overall, it can be summarized that the combination therapies presented better effectiveness in all cell lines compared to their individual therapy counterparts. In the viability assay, the tamoxifen-resistant cell lines showed stronger viability-inhibiting effects on all combination treatments compared to the parental cell lines. It was also observed that the combined inhibition of metabolism was superior to the combined tamoxifen therapy in all cell lines. The combination of the two metabolism inhibitors 2-DG and CB-839 showed a significantly stronger inhibition of cell viability in all four cell lines compared to the individual treatments. Through the triple combination therapy, a synergistic-supportive inhibitory effect could be produced. In the viability assay alone, therapy with low-dose 4-OHT showed a significantly better viability-inhibiting effect in the tamoxifen-resistant cell lines when combined with CB-839 compared to the respective individual treatments. The combination of 4-OHT and 2-DG failed with no significantly stronger inhibition of viability in any of the four cell lines.

In order to consider potential effects on healthy breast tissue, studies were also carried out with the normal breast epithelial cell line MCF-10A (not shown). Whereas these cells exhibit a non-significant inhibitory effect of 004-OHT on cell growth, the anti-metabolic agents 2-DG and CB-839 alone or in combination did not show any effects. Furthermore, there was no change in the effects of 4-OHT if the cells were treated in combination with 2-DG and/or CG-839. Tamoxifen has been reported to have beneficial effects on normal breast tissue. Tamoxifen affects the normal breast epithelium and its adhesiveness, which probably contributes to the clinically observed decrease of breast density and thus could lead to a potentially reduced risk of developing breast tumors [29]. The research group around Daurio et al. examined the action mechanism of tamoxifen on various breast cancer cell lines. They found that tamoxifen had an inhibitory effect on mitochondrial-complex-I, which led to an increase in the AMP/ATP ratio, resulting in stronger glycolysis via the activation of AMPK. They concluded that a combination of tamoxifen and a glycolysis inhibitor was ideal [30]. This could not be confirmed in our work using the combination of 4-OHT and 2-DG; none of the four cell lines examined showed a significantly better effect on viability inhibition under the combination therapy compared to the individual treatments. However, it must be noted that this work was carried out with comparatively low concentrations. Furthermore, Ambrosio et al. found that glucose inhibits tamoxifen sensitivity via CTFG (connective tissue growth factor) [31]. Attia et al. also showed that tamoxifen cytotoxicity could be increased in vitro in MCF7 and T47D cell lines by combining it with the glycolysis inhibitor 3-bromopyruvate [32]. In this work, no significant cytotoxic optimization could be shown under the combination treatment of tamoxifen and the glycolysis inhibitor in the here used low concentrations. The working group around Woo et al. was able to use MCF7-TR cells to demonstrate, that the glycolysis activity, Akt/mTOR and HIF-1-alpha were increased compared to the tamoxifen-sensible cells. As a consequence, they recommended glycolysis inhibition for hormone-resistant breast carcinoma cells [33]. In the viability assay in this work, the tamoxifen-resistant cell lines showed an earlier, but not as strong effect compared to the parental lines on the individual treatment with 2-DG. Nonetheless, they showed stronger viability-inhibiting effects on all combination treatments compared to the parental cell lines, with the combination treatments with the glycolysis inhibitor CB-839 in particular achieving better effects compared to 2-DG.

To better classify the inhibition of viability with regard to a more precise mechanism among the various treatments, an apoptosis assay was carried out. It could be shown that to a large extent, there was a correlation between the viability assay and apoptosis. In the MCF7 cell line, inhibition of the mitochondrial membrane potential was observed under the triple combination, but also under the combinations of 4-OHT and 2-DG as well as the metabolism inhibitors. The same was found in the associated tamoxifen-resistant MCF7-TR cell line, as well as the T47D and T47D-TR cell lines. It was observed that in three of the four cell lines (MCF7, T47D, T47D-TR) a significantly strong inhibition of the mitochondrial membrane potential was caused by the combination treatment 4-OHT + 2-DG at the time of measurement was performed. This effect of apoptosis induction was not reflected in the viability behavior and is most likely to be explained by the sensitivity of the method itself.

To investigate a possible connection between the effects of the various anti-metabolic treatments on cell viability and the role of the oncogenic protein c-Myc in the regulation and control of cancer cell metabolism [25], expression of c-Myc was analyzed. C-Myc belongs to the Myc family and, as a transcription factor, plays a central role in relation to transcription, transformation, proliferation and apoptosis. Based on our results, the role of c-Myc in particular for cell viability was examined. C-Myc can be activated by hormones, such as estrogen, and growth factors (GF) [34,35]. In addition, other second messengers such as Ras (Rat sarcoma) play a role in the activation of Myc [36]. If Myc is triggered, it leads to cell proliferation by stimulating the cell cycle and activating cyclins as well as by inhibiting growth inhibitors [37]. On the other hand, Myc promotes apoptosis by activating ARF (adenosyl ribosylation factor) and p53 and by inhibiting anti-apoptotic proteins [38,39]. This equilibrium of proliferation and induction of apoptosis initially represents a barrier to tumor development.

However, if various processes such as a p53 mutation or the anti-apoptotic protein Bcl-2 lead to an inhibition of apoptosis, the system is out of balance and tumor development is guaranteed [40]. It is proven, that in a large number of malignant tumors Myc is therefore upregulated [41]. A knock-down of Myc led to an inhibition of proliferation in malignant tumors, where Myc was previously upregulated, but also to an increase in apoptosis, which suggests the dependence of malignant tumors on c-Myc [42]. Regarding therapeutic targets, it has previously been shown that in cells presenting an upregulation of c-Myc, glucose or glutamine withdrawal led to increased apoptosis, as insufficient metabolites were available for the increased proliferation induced by Myc [43,44]. The active ingredients used are therefore in the context of c-Myc. 4-OHT as SERM regulates the ERs and thereby leads to the inhibition of c-Myc and thus to the inhibition of proliferation. 2-DG as a glycolysis inhibitor and CB-839 as a glutaminase inhibitor intervene in the metabolism and decrease the viability increased by c-Myc, which leads to increased apoptosis.

Watson et al. have already demonstrated c-Myc amplification in MCF7 and T47D cell lines [45]. In our work, the involvement of c-Myc in MCF7, MCF7-TR, T47D and T47D-TR could also be observed. A work by McNeal et al. showed that c-Myc plays a central role in the development of endocrine resistance [46]. The upregulation of c-Myc is associated with a secondary development of resistance [47]. It was also shown that tamoxifen-resistant cell lines have higher c-Myc levels compared to non-resistant cell lines, combined with more invasive and proliferative growth and therefore poorer prognosis [48]. In addition, an upregulation of glucose metabolism associated with c-Myc was found in cell lines with resistance to endocrine therapy [49]. Fallah et al. even recommended upregulating Myc as a predictor of antiestrogen therapy resistance [50]. It could be consistently confirmed by our results: in comparison to the parental cell lines MCF7 and T47D, a stronger expressivity of c-Myc was observed in the tamoxifen-resistant cell lines MCF7-TR and T47D-TR.

We could also show that c-Myc downregulation was partially significant under almost all treatments, especially under the combination treatments. The fact that suppression of c-Myc expression using specific siRNA leads to the loss of tamoxifen-resistance in tamoxifen-resistant cell lines MCF7-TR and T47D-TR supports the important role of c-Myc in this context. This may confirm the connection between the active ingredients and c-Myc. Treatment with 4-OHT alone was an exception in the case of the tamoxifen-resistant cell lines; there was no down-regulation of the c-Myc. This shows that the secondary tamoxifen resistance must have an impact on c-Myc expression.

## 5. Conclusions

Combination treatments of 4-OHT with CB-839, with 2-DG and CB-839 as well as the triple combination of 4-OHT, 2-DG and CB-839 have significantly stronger inhibitory effects in vitro compared to the individual treatments with 4-OHT, 2-DG or CB-839 alone. However, none of the cell lines showed a therapy optimization compared to the individual treatments with the combination of 4-OHT and 2-DG. The viability-inhibiting effects were largely reflected in the induction of apoptosis. In addition, a downregulation of the proto-oncogene c-Myc could be observed during the treatments. Furthermore, after the suppression of c-Myc expression using specific siRNA the loss of tamoxifen-resistance of the tamoxifen-resistant BC cells was observed. These results support the major role of c-Myc in the regulation of tamoxifen resistance and cancer metabolism. The cell lines MCF7 and T47D, due to their properties as ER- and PR-positive epithelial, non-invasive cell lines, represent an in vitro model of breast cancer of the subtype luminal A and thus the most common form of BC. In practice, tamoxifen is an established therapy recommendation for this subtype. However, therapy becomes more difficult due to the development of side effects and occurrences of secondary resistance. In this work, it was shown that the therapy with tamoxifen alone in the cell lines MCF7 and T47D could be optimized by combination with the glutaminase inhibitor CB-839. In vivo, it would make sense to evaluate this combination, especially with the purpose of reducing the concentrations of the individual substances in order to counteract the possible development of side effects. The development of secondary tamoxifen resistance is a common problem in practice. With the tamoxifen-resistant cell lines MCF7-TR and T47D-TR in connection with their parental lines, an in vitro model was available to compare the different treatments. It could be shown that the tamoxifen-resistant cell lines in particular reacted more sensitively to the combined metabolic inhibition in comparison to their parental cell lines. An in vivo evaluation of this combination would be valuable. With the T47D-TR cell line, a significantly stronger effect compared to all other treatments was observed even using the triple combination treatment. Therefore, it would be interesting to evaluate whether tamoxifen resistance could be overcome by treatment with the combination of both inhibitors.

## Data Availability

The datasets used and/or analyzed during the current study are available from the corresponding author on reasonable request.

## Supplementary Materials

The following are available online at https://www.mdpi.com/article/10.3390/cells10092398/s1, Table S1: Viability of human breast cancer cell lines MCF7, T47D and their tamoxifen-resistant sublines MCF7-TR and T47D-TR after treatment without or with 4-OHT, 2-DG or CB-839, Table S2: Viability of human breast cancer cell lines MCF7, T47D and their tamoxifen-resistant sublines MCF7-TR and T47D-TR after treatment without or with 4-OHT, 2-DG or CB-839 or different combinations, Table S3: Mitochondrial membrane potential of human breast cancer cell lines MCF7, T47D and their tamoxifen-resistant sublines MCF7-TR and T47D-TR after treatment without or with 4-OHT, 2-DG or CB-839 or different combinations, Table S4: Viability of human breast cancer cell lines MCF7 (C), T47D (D) and their tamoxifen-resistant sublines MCF7-TR and T47D-TR after c-Myc suppression using specific siRNA. Effect of c-Myc knock down on tamoxifen efficacy on the viability of human breast cancer cell lines MCF7, T47D and their tamoxifen-resistant sublines MCF7-TR and T47D-TR, Table S5: Protein expression of c-Myc in human breast cancer cell lines MCF7, T47D and their tamoxifen-resistant sublines MCF7-TR (B) and T47D-TR (D) after treatment without or with 4-OHT, 2-DG or CB-839 or different combinations.
