# Supplementary material for: Inhibition of Metabolism as a Therapeutic Option for Tamoxifen-Resistant Breast Cancer Cells
Source: Cells. 2021 Sep 12;10(9):2398. doi: 10.3390/cells10092398 (PMC8467413; doi:10.3390/cells10092398)
Supplement: Supplementary file 1 [file cells-10-02398-s001.zip › cells-1316232-Sl.pdf]

Supplement to

Inhibition of metabolism as a therapeutic option for tamoxifen-resistant breast cancer cells.

Friederike Steifensand[1], Julia Gallwas[1], Gerd Bauerschmitz[1+], Carsten Gründker[1+*]

1 Department of Gynecology and Obstetrics, University Medicine Göttingen, Göttingen, Germany; f.steifensand@stud.uni-goettingen.de (F.S.); julia.gallwas@med.uni-goettingen.de (J.G.); gerd.bauerschmitz@med.uni-goettingen.de (G.B.)
* Correspondence: grundker@med.uni-goettingen.de; Tel.: +49-551-3969810
\+ CG and GB contributed equally to this work

Table S1. Viability of human breast cancer cell lines MCF7, T47D and their tamoxifen-resistant sublines MCF7-TR and T47D-TR after treatment without or with 4-OHT, 2-DG or CB-839.

**MCF7 (Fig. 1A)**	**%**	**SEM**	**N**	**Significant Differences**
Control	100.00	0.00	3	
100 nM 4-OHT	106.33	3.04	3	
1 µM 4-OHT	103.70	0.34	3	
5 µM 4-OHT	74.90	4.61	3	p < 0.05 vs. control
0.625 mM 2-DG	99.35	4.43	3	
2.5 mM 2-DG	94.18	0.73	3	
5 mM 2-DG	60.73	0.25	3	p <0.001 vs. control, p <0.05 vs. 100nM 4-OHT, p <0.01 vs. 0.625 mM 2-DG, p <0.001 vs. 1 µM CB-839, p <0.01 vs. 5 µM CB-839
1 µM CB-839	97.85	0.54	3	
5 µM CB-839	93.04	0.98	3	
10 µM CB-839	84.37	0.41	3	
**MCF7-TR (Fig. 1B)**	**%**	**SEM**	**N**	**Significant Differences**
Control	100.00	0.00	3	
100 nM 4-OHT	100.48	0.25	3	
1 µM 4-OHT	98.89	1.80	3	
5 µM 4-OHT	99.87	1.54	3	
0.625 mM 2-DG	98.78	1.38	3	
2.5 mM 2-DG	69.88	1.10	3	p <0.0001 vs. control, p <0.0001 vs. 100nM 4-OHT, p <0.0001 vs. 1 µM 4-OHT, p <0.0001 vs. 5 µM 4-OHT, p <0.0001 vs. 0.625 mM 2-DG, p <0.0001 vs. 1 µM CB-839, p <0.0001 vs. 5 µM CB-839
5 mM 2-DG	65.46	1.75	3	p <0.0001 vs. control, p <0.0001 vs. 100nM 4-OHT, p <0.0001 vs. 1 µM 4-OHT, p <0.0001 vs. 5 µM 4-OHT, p <0.0001 vs. 0.625 mM 2-DG, p <0.0001 vs. 1 µM CB-839, p <0.0001 vs. 5 µM CB- 839
1 µM CB-839	106.63	4.39	3	
5 µM CB-839	96.63	1.27	3	

10 μM CB-839	65.07	2.83	3	p <0.0001 vs. control, p <0.0001 vs. 100nM 4-OHT, p <0.0001 vs. 1 μM 4-OHT, p <0.0001 vs. 5 μM 4-OHT, p <0.0001 vs. 0.625 mM 2-DG, p <0, 0001 vs. 1 μM CB-839, p <0.0001 vs. 5 μM CB-839
**T47D (Fig. 1C)**	**%**	**SEM**	**N**	**Significant Differences**
Control	100.00	0.00	3	
100 nM 4-OHT	96.67	2.45	3	
1 μM 4-OHT	99.26	4.25	3	
5 μM 4-OHT	61.45	1.86	3	p <0.0001 vs. control; p <0.0001 vs. 100nM 4-OHT; e, p <0.0001 vs. 1 μM 4-OHT; p <0.0001 vs. 0.625 mM 2-DG; p <0.0001 vs. 2.5 mM 2-DG; p <0.0001 vs. 1 μM CB-839; p <0.0001 vs. 5 μM CB-839;, p <0.01 vs. 10 μM CB-839
0.625 mM 2-DG	107.16	5.99	3	
2.5 mM 2-DG	99.32	2.18	3	
5 mM 2-DG	51.53	2.59	3	p <0.0001 vs. control; p <0.0001 vs. 100nM 4-OHT; e, p <0.0001 vs. 1 μM 4-OHT; p <0.0001 vs. 0.625 mM 2-DG; p <0.0001 vs. 2.5 mM 2-DG; p <0.0001 vs. 1 μM CB-839; p <0.0001 vs. 5 μM CB-839; p <0.0001 vs. 10 μM CB-839
1 μM CB-839	98.13	0.35	3	
5 μM CB-839	91.90	0.60	3	p <0.05 vs. 0.625 mM 2-DG; 10 μM CB-839: 80.93 ± 0.8768%, p <0.01 vs. control; p <0.05 vs. 100 nM 4-OHT; p <0.01 vs. 1 μM 4-OHT; p <0.0001 vs. 0.625 mM 2-DG; p <0.01 vs. 2.5 mM 2-DG; p <0 , 01 vs. 1 μM CB-839
10 μM CB-839	80.93	0.88	3	
**T47D-TR (Fig. 1D)**	**%**	**SEM**	**N**	**Significant Differences**
Control	100.00	0.00	3	
100 nM 4-OHT	97.58	2.01	3	
1 μM 4-OHT	98.23	4.90	3	
5 μM 4-OHT	89.15	0.51	3	p <0.05 vs. control
0.625 mM 2-DG	91.03	2.02	3	
2.5 mM 2-DG	80.35	1.64	3	p <0.0001 vs. control; p <0.001 vs. 100 nM 4-OHT; p <0.001 vs. 1 μM 4-OHT; p <0.05 vs. 0.625 mM 2-DG; p <0.01 vs. 1 μM CB-839
5 mM 2-DG	75.05	1.84	3	p <0, 0001 vs. control; p <0.0001 vs. 100nM 4-OHT; p <0.0001 vs. 1 μM 4-OHT; p <0.01 vs. 5 μM 4-OHT; p <0.001 vs. 0.625 mM 2-DG; p <0.0001 vs. 1 μM CB-839; p <0.01 vs. 5 μM CB-839
1 μM CB-839	94.10	1.20	3	
5 μM CB-839	88.83	0.37	3	
10 μM CB-839	80.93	0.61	3	p <0.0001 vs. control; p <0.001 vs. 100nM 4-OHT; p <0.001 vs. 1 μM 4-OHT; p <0.05 vs. 0.625 mM 2-DG; p <0.01 vs. 1 μM CB-839

Table S2. Viability of human breast cancer cell lines MCF7 (A), T47D (C) and their tamoxifen-resistant sublines MCF7-TR (B) and T47D-TR (D) after treatment without or with 4-OHT, 2-DG or CB-839 or different combinations.

**MCF7 (Fig. 2A)**	**%**	**SEM**	**N**	**Significant Differences**
Control	100.00	0.00	3	
1 μM 4-OHT	103.70	0.34	3	
2.5 mM 2-DG	94.18	0.73	3	
5 μM CB-839	93.04	0.98	3	
1 μM 4-OHT + 2.5 mM 2-DG	90.36	1.34	3	p <0.05 vs. control; p <0.001 vs. 1 μM 4-OHT
1 μM 4-OHT + 5 μM CB-839	87.31	1.59	3	p <0.01 vs. control; p <0.0001 vs. 1 μM 4-OHT
2.5 mM 2-DG + 5 μM CB-839	76.74	1.52	3	p <0.0001 vs. control; p <0.0001 vs. 1 μM 4-OHT; p <0.0001 vs. 2.5 mM 2-DG; p <0.001 vs. 5 μM CB-839; p <0.001 vs. 1 μM 4-OHT + 2.5 mM 2-DG; p <0.01 vs. 1 μM 4-OHT + 5 μM CB-839
1 μM 4-OHT + 2.5 mM 2-DG + 5 μM CB-839	70.36	3.90	3	p <0.0001 vs. control; p <0.0001 vs. 1 μM 4-OHT; p <0.0001 vs. 2 , 5 mM 2-DG; p <0.0001 vs. 5 μM CB-839; p <0.0001 vs. 1 μM 4-OHT + 2.5 mM 2-DG; p <0.0001 vs. 1 μM 4-OHT + 5 μM CB-839
**MCF7-TR (Fig. 2B)**	**%**	**SEM**	**N**	**Significant Differences**
Control	100.00	0.00	3	
1 μM 4-OHT	98.89	1.80	3	
2.5 mM 2-DG	69.88	1.10	3	p <0.0001 vs. control; p <0.0001 vs. 1 μM 4-OHT; p <0.0001 vs. 5 μM CB-839; p <0.01 vs. 1 μM 4-OHT + 5 μM CB-839
5 μM CB-839	96.63	1.27	3	
1 μM 4-OHT + 2.5 mM 2-DG	72.20	3.43	3	p <0.0001 vs. control; p <0.0001 vs. 1 μM 4-OHT; p < 0.0001 vs. 5 μM CB-839; p <0.05 vs. 1 μM 4-OHT + 5 μM CB-839
1 μM 4-OHT + 5 μM CB-839	80.80	0.69	3	p <0.0001 vs. control; p <0.0001 vs. 1 μM 4-OHT; p <0.001 vs. 5 μM CB-839
2.5 mM 2-DG + 5 μM CB-839	63.53	1.67	3	p <0.0001 vs. control; p <0.0001 vs. 1 μM 4- OHT; p <0.0001 vs. 5 μM CB-839; p <0.05 vs. 1 μM 4-OHT + 2.5 mM 2-DG; p <0.0001 vs. 1 μM 4-OHT + 5 μM CB-839
1 μM 4-OHT + 2.5 mM 2-DG + 5 μM CB-839	61.61	1.55	3	p <0.0001 vs. control; p <0.0001 vs. 1 μM 4-OHT; p <0.0001 vs. 5 μM CB-839; p <0.001 vs. 1 μM 4-OHT + 2.5 mM 2-DG; p <0.0001 vs. 1 μM 4-OHT + 5 μM CB-839
**T47D (Fig. 2C)**	**%**	**SEM**	**N**	**Significant Differences**
Control	100.00	0.00	3	
1 μM 4-OHT	98.33	0.91	3	
2.5 mM 2-DG	88.24	0.73	3	p <0.001 vs. control; p <0.01 vs. 1 μM 4-OHT.T47D, 5 μM CB-839: 91.14 ± 0.7267%, p <0.01 vs. control; p <0.05 vs. 1 μM 4-OHT
5 μM CB-839	91.14	0.73	3	
1 μM 4-OHT + 2.5 mM 2-DG	91.19	2.48	3	p <0.05 vs. 1 μM 4-OHT
1 μM 4-OHT + 5 μM CB-839	88.55	0.69	3	p < 0.001 vs. control; p <0.01 vs. 1 μM 4-OHT

2.5 mM 2-DG + 5 μM CB-839	75.99	1.80	3	p <0.0001 vs. control; p <0.0001 vs. 1 μM 4-OHT; p <0.001 vs. 2.5 mM 2-DG; p <0.0001 vs. 5 μM CB-839; p <0.0001 vs. 1 μM 4-OHT + 2.5 mM 2-DG; p <0.001 vs. 1 μM 4-OHT + 5 μM CB-839
1 μM 4-OHT + 2.5 mM 2-DG + 5 μM CB-839	73.18	1.80	3	p <0.0001 vs. control; p <0.0001 vs. 1 μM 4-OHT; p <0.0001 vs. 2.5 mM 2-DG; p <0.0001 vs. 5 μM CB-839; p <0.0001 vs. 1 μM 4-OHT + 2, 5 mM 2-DG; p <0.0001 vs. 1 μM 4-OHT + 5 μM CB-839
**T47D-TR (Fig. 2D)**	**%**	**SEM**	**N**	**Significant Differences**
Control	100.00	0.00	3	
1 μM 4-OHT	98.23	4.90	3	
2.5 mM 2-DG	80.35	1.64	3	p <0.01 vs. control; p <0.01 vs. 1 μM 4-OHT
5 μM CB-839	88.83	0.37	3	
1 μM 4-OHT + 2.5 mM 2-DG	88.02	2.48	3	
1 μM 4-OHT + 5 μM CB-839	84.08	2.70	3	p <0.05 vs. control; p <0.05 vs. 1 μM 4-OHT
2.5 mM 2-DG + 5 μM CB-839	65.36	2.68	3	p <0.0001 vs. control; p <0.0001 vs. 1 μM 4-OHT; p <0, 05 vs. 2.5 mM 2-DG; p <0.001 vs. 5 μM CB-839; p <0.001 vs. 1 μM 4-OHT + 2.5 mM 2-DG; p <0.01 vs. 1 μM 4-OHT + 5 μM CB-839
1 μM 4-OHT + 2.5 mM 2-DG + 5 μM CB-839	50.42	3.69	3	p <0.0001 vs. control; p <0.0001 vs. 1 μM 4-OHT; p <0.0001 vs. 2.5 mM 2-DG; p <0.0001 vs. 5 μM CB- 839; p <0.0001 vs. 1 μM 4-OHT + 5 μM CB-839; p <0.05 vs. 2.5 mM 2-DG + 5 μM CB-839

Table S3. Mitochondrial membrane potential of human breast cancer cell lines MCF7 (A), T47D (C) and their tamoxifen-resistant sublines MCF7-TR (B) and T47D-TR (D) after treatment without or with 4-OHT, 2-DG or CB-839 or different combinations.

**MCF7 (Fig. 3A)**	**%**	**SEM**	**N**	**Significant Differences**
Control	1.00	0.00	5	
1 μM 4-OHT	0.89	0.09	5	
2.5 mM 2-DG	0.75	0.06	5	
5 μM CB-839	0.80	0.08	5	
1 μM 4-OHT + 2.5 mM 2-DG	0.57	0.05	5	p <0.001 vs. control; p <0.05 vs. 1 μM 4-OHT
1 μM 4-OHT + 5 μM CB-839	0.75	0.07	5	
2.5 mM 2-DG + 5 μM CB-839	0.62	0.06	5	p <0.01 vs. control
1 μM 4-OHT + 2.5 mM 2-DG + 5 μM CB-839	0.53	0.06	5	p <0.001 vs. control; p <0.01 vs. 1 μM 4-OHT
**MCF7-TR (Fig. 3B)**	**%**	**SEM**	**N**	**Significant Differences**
Control	1.00	0.00	5	
1 μM 4-OHT	0.78	0.04	5	
2.5 mM 2-DG	0.88	0.11	5	
5 μM CB-839	0.70	0.05	5	
1 μM 4-OHT + 2.5 mM 2-DG	0.66	0.05	5	p <0.05 vs. control
1 μM 4-OHT + 5 μM CB-839	0.65	0.07	5	
2.5 mM 2-DG + 5 μM CB-839	0.56	0.10	5	p <0.01 vs. control
1 μM 4-OHT + 2.5 mM 2-DG + 5 μM CB-839	0.51	0.10	5	p <0.01 vs. control; p <0.05 vs. 2.5 mM 2-DG
**T47D (Fig. 3C)**	**%**	**SEM**	**N**	**Significant Differences**
Control	1.00	0.00	5	
1 μM 4-OHT	0.69	0.03	5	p <0.001 vs. control
2.5 mM 2-DG	0.87	0.02	5	
5 μM CB-839	0.77	0.05	5	
1 μM 4-OHT + 2.5 mM 2-DG	0.56	0.02	5	p <0.0001 vs. control; p <0.001 vs. 2.5 mM 2-DG; p <0.05 vs. 1 μM 4- OHT + 5 μM CB-839
1 μM 4-OHT + 5 μM CB-839	0.85	0.11	5	
2.5 mM 2-DG + 5 μM CB-839	0.69	0.06	5	p <0.001 vs. control
1 μM 4-OHT + 2.5 mM 2-DG + 5 μM CB-839	0.65	0.06	5	p <0.001 vs. control
**T47D-TR (Fig. 3D)**	**%**	**SEM**	**N**	**Significant Differences**
Control	1.00	0.00	5	
1 μM 4-OHT	0.73	0.02	5	p <0.01 vs. control
2.5 mM 2-DG	0.70	0.04	5	p <0.01 vs. control; p <0.05 vs. 5 μM CB-839
5 μM CB-839			5	

1 μM 4-OHT + 2.5 mM 2-DG	0.50	0.01	5	p <0.0001 vs. control; p <0.05 vs. 1 μM 4-OHT; p <0.0001 vs. 5 μM CB-839; p <0.0001 vs. 1 μM 4-OHT + 5 μM CB-839
1 μM 4-OHT + 5 μM CB-839	0.88	0.04	5	
2.5 mM 2-DG + 5 μM CB-839	0.65	0.03	5	p <0.001 vs. control; p <0.01 vs. 5 μM CB-839; p <0.05 vs. 1 μM 4-OHT + 5 μM CB-839
1 μM 4-OHT + 2.5 mM 2-DG + 5 μM CB-839	0.65	0.03	5	p <0.0001 vs. control; p <0.001 vs. 5 μM CB -839; p <0.01 vs. 1 μM 4-OHT + 5 μM CB-839

Table S4. Viability of human breast cancer cell lines MCF7 (C), T47D (D) and their tamoxifen-resistant sublines MCF7-TR (C) and T47D-TR (D) after c-Myc suppression using specific siRNA. Effect of c-Myc knock down on tamoxifen efficacy on the viability of human breast cancer cell lines MCF7 (E), T47D (F) and their tamoxifen-resistant sublines MCF7-TR (E) and T47D-TR (F).

**MCF7 (Fig. 4C)**	%	**SEM**	**N**	**Significant Differences**
MCF7	100.00	2.32	6	
MCF7 c-Myc⁻	93.17	3.18	6	
MCF7-TR	100.00	1.81	6	
MCF7-TR c-Myc⁻	86.70	2.79	6	p <0.01 vs. MCF7-TR control (= 100%)
**MCF7-TR (Fig. 4D)**	%	**SEM**	**N**	**Significant Differences**
T47D	100.00	2.03	6	
T47D c-Myc⁻	90.62	3.22	6	T47D: 90.62 ± 3.22%, N = 6
T47D-TR	100.00	4.07	6	
T47D-TR c-Myc⁻	75.48	3.32	6	p <0.0001 vs. T47D-TR control (= 100%)
**T47D (Fig. 4E)**	%	**SEM**	**N**	**Significant Differences**
MCF7	100.00	0.00	3	
MCF7 + 5 μM 4-OHT	74.90	4.61	3	p <0.001 vs. untreated control (= 100%), p<0.001 vs. treated MCF7-TR
MCF7-TR + 5 μM 4-OHT	99.86	1.54	3	
MCF7-TR c-Myc⁻ + 5 μM 4-OHT	71.87	2.12	3	p<0.001 vs. untreated control (=100%), p<0.001 vs. treated MCF7-TR
**T47D-TR (Fig. 4F)**	%	**SEM**	**N**	**Significant Differences**
T47D	100.00	0.00	3	
T47D + 5 μM 4-OHT	61.45	1.86	3	p <0.0001 vs. untreated control (= 100%), p<0.001 vs. treated T47D-TR
T47D-TR + 5 μM 4-OHT	89.15	0.51	3	
T47D-TR c-Myc⁻ + 5 μM 4-OHT	51.83	4.82	3	p<0.0001 vs. untreated control (=100%), p<0.0001 vs. treated T47D-TR

Table S5. Protein expression of c-Myc in human breast cancer cell lines MCF7 (A), T47D (C) and their tamoxifen-resistant sublines MCF7-TR (B) and T47D-TR (D) after treatment without or with 4-OHT, 2-DG or CB-839 or different combinations.

**MCF7 (Fig. 5A)**	**%**	**SEM**	**N**	**Significant Differences**
Control	100.00	0.00	4	
1 μM 4-OHT	15.30	3.99	4	p <0.0001 vs. control
2.5 mM 2-DG	44.76	17.06	4	p <0.01 vs. control
5 μM CB-839	21.42	2.66	4	p <0.001 vs. control
1 μM 4-OHT + 2.5 mM 2-DG	34.86	18.03	4	p <0.01 vs. control
1 μM 4-OHT + 5 μM CB-839	14.46	4.11	4	p <0.0001 vs. control
2.5 mM 2-DG + 5 μM CB-839	33.26	8.49	4	p <0.01 vs. control
1 μM 4-OHT + 2.5 mM 2-DG + 5 μM CB-839	7.54	3.27	4	p <0.0001 vs. control
**MCF7-TR (Fig. 5B)**	**%**	**SEM**	**N**	**Significant Differences**
Control	100.00	0.00	3	
1 μM 4-OHT	111.33	15.43	3	
2.5 mM 2-DG	75.35	7.75	3	
5 μM CB-839	56.29	14.67	3	
1 μM 4-OHT + 2.5 mM 2-DG	40.33	15.96	3	p <0.05 vs. control; p <0.05 vs. 1 μM 4-OHT
1 μM 4-OHT + 5 μM CB-839	27.67	5.55	3	p <0.05 vs. control; p <0.001 vs. 1 μM 4-OHT
2.5 mM 2-DG + 5 μM CB-839	36.67	13.20	3	p <0.05 vs. control; p <0.001 vs. 1 μM 4-OHT
1 μM 4-OHT + 2.5 mM 2-DG + 5 μM CB-839	27.67	13.17	3	p <0.05 vs. control; p <0.001 vs. 1 μM 4-OHT
**T47D (Fig. 5C)**	**%**	**SEM**	**N**	**Significant Differences**
Control	100.00	0.00	3	
1 μM 4-OHT	48.60	13.04	3	
2.5 mM 2-DG	74.40	15.54	3	
5 μM CB-839	71.19	27.51	3	
1 μM 4-OHT + 2.5 mM 2-DG	71.60	24.39	3	
1 μM 4-OHT + 5 μM CB-839	14.60	6.19	3	p <0, 01 vs. control
2.5 mM 2-DG + 5 μM CB-839	31.80	10.57	3	
1 μM 4-OHT + 2.5 mM 2-DG + 5 μM CB-839	22.60	3.67	3	p <0.01 vs. control
**T47D-TR (Fig. 5D)**	**%**	**SEM**	**N**	**Significant Differences**
Control	100.00	0.00	3	
1 μM 4-OHT	107.94	22.60	3	
2.5 mM 2-DG	41.01	28.51	3	
5 μM CB-839	11.93	4.11	3	p <0.01 vs. 1 μM 4-OHT
1 μM 4-OHT + 2.5 mM 2-DG	35.64	16.77	3	
1 μM 4-OHT + 5 μM CB-839	26.61	11.15	3	p <0.05 vs. 1 μM 4-OHT

2.5 mM 2-DG + 5 μM CB-839	9.37	5.89	3	$p < 0.05$ vs. control; $p < 0.01$ vs. 1 μM 4-OHT
1 μM 4-OHT + 2.5 mM 2-DG + 5 μM CB-839	21.98	16.99	3	$p < 0.05$ vs. 1 μM 4-OHT
